# Clinical Evaluation of the Progression of Liver Disease in Patients Coinfected With HBV and HDV in the Western Amazon Region of Brazil

**DOI:** 10.1155/ijh/2054487

**Published:** 2025-06-05

**Authors:** Júlia Teixeira Ton, Ana Maísa Passos-Silva, Ester Teixeira Ton, Eugênia de Castro Silva, Alcione Oliveira Santos, Adrhyan Araújo, Deusilene Vieira, Juan Miguel Villalobos Salcedo, Mariana Pinheiro Alves Vasconcelos

**Affiliations:** ^1^Centro de Pesquisa em Medicina Tropical de Rondônia (CEPEM), Porto Velho, Brazil; ^2^Centro Universitário São Lucas (UNISL), Porto Velho, Brazil; ^3^Laboratório de Virologia Molecular, Fundação Oswaldo Cruz de Rondônia (FIOCRUZ/RO), Porto Velho, Brazil; ^4^Ambulatório Especializado em Hepatites Virais de Rondônia (AHV/RO), Porto Velho, Brazil; ^5^Programa de Pós-graduação em Biologia Experimental, Universidade Federal de Rondônia (UNIR-FIOCRUZ/RO), Porto Velho, Rondônia, Brazil; ^6^Departamento de Medicina, Universidade Federal de Rondônia (UNIR/RO), Porto Velho, Rondônia, Brazil; ^7^Centro de Medicina Tropical de Rondônia (CEMETRON), Porto Velho, Brazil

**Keywords:** fibrosis, hepatitis B, hepatitis delta

## Abstract

**Background:** Worldwide, an estimated 296 million individuals are chronic carriers of hepatitis B virus (HBV), with approximately 5% also coinfected with hepatitis delta virus (HDV). In Brazil, HBV and HDV are endemic in the states of the Western Amazon. This study is aimed at characterizing a cohort of patients coinfected with HBV and HDV and comparing their clinical and epidemiological profiles with those of HBV monoinfected individuals.

**Methods:** The study involved a retrospective clinical analysis of individuals monoinfected with HBV and coinfected with HDV, conducted between 2017 and 2018 in Rondônia, Brazil.

**Results:** A total of 324 patients were enrolled in the study, comprising 302 individuals with HBV monoinfection and 22 with HBV–HDV coinfection. Patients with HDV exhibited significantly more clinical signs of advanced liver disease. Using APRI and FIB-4 scores with cut-off values established for HBV, over 40% of HDV-infected patients had values indicative of advanced liver fibrosis, compared to 5%–10% in the HBV monoinfected group. Across all evaluated parameters of liver disease, HDV patients displayed more severe characteristics, with 45.5% already showing signs of advanced liver disease at the time of enrollment.

**Conclusion:** Our study underscores the importance of the clinical analysis of hepatitis delta as a more aggressive disease model compared to hepatitis B in the population of the Western Brazilian Amazon, highlighting its significance as a public health concern in the region.

## 1. Background

Hepatitis B is a significant global public health challenge, with an estimated 296 million individuals worldwide living as chronic carriers of the hepatitis B virus (HBV) [1, 2]. The global prevalence of hepatitis delta virus (HDV) remains a subject of debate; however, it is estimated that approximately 5% of individuals with chronic HBV infection are coinfected with HDV [2].

HBV is primarily transmitted through contact with the blood or bodily fluids of an infected individual [3, 4]. In highly endemic regions, such as the Western Amazon, HBV is predominantly transmitted through horizontal transmission via intrafamily contact or vertically from mother to child during childbirth [1, 5, 6]. HDV is primarily transmitted through parenteral routes, such as exposure to contaminated blood or needles, and through sexual contact, both of which significantly contribute to the spread of the disease [7, 8]. Hepatitis delta can be acquired through coinfection or superinfection, with the latter being most strongly associated with severe outcomes. In cases of superinfection, liver cirrhosis develops in 70% of patients within 5–10 years, a rate three times higher than that observed in HBV monoinfection [9, 10]; however, the pathological progression of the disease can be influenced by various factors, including virological, immunological, and genetic characteristics of the affected individuals [11–13].

In Brazil, despite the introduction of a nationwide HBV vaccination program for children by the National Immunization Program (PNI) in 1998, HBV prevalence remains a significant public health concern, particularly in specific endemic regions [14] later extended to adults in 2013 [15]; both HBV and HDV remain endemic in the Amazon region, with high detection rates of both viruses reported across the country [16–18]; over the past 23 years, among the 289,029 reported cases of hepatitis B in Brazil, the capitals of Rondônia and Acre have consistently recorded the highest incidence rates nationwide. Furthermore, hepatitis delta cases are heavily concentrated in the north region, accounting for 72.5% (3,281) of the total cases reported during this period [19]. However, the clinical profile of these individuals remains inadequately characterized. Given the high endemicity of these diseases in the region, this study is aimed at investigating the clinical and epidemiological dynamics of HBV monoinfected and HDV coinfected individuals in the Western Amazon region of Brazil.

## 2. Methods

### 2.1. Ethics Declarations

The study was approved by the Ethics Committee of the Tropical Medicine Research Center (CEPEM/RO) (3.826.726), and written informed consent was obtained from all participants.

### 2.2. Type and Location of the Study

This is a retrospective, observational, cross-sectional study conducted at the Ambulatório Especializado em Hepatites Virais (AHV/RO) of Centro de Pesquisa em Medicina Tropical do Estado de Rondônia (CEPEM/RO), Brazil.

### 2.3. Study Population

The study population comprised 324 individuals who were already under clinical monitoring at AHV/RO between 2017 and 2018, stratified into two groups: 302 individuals with HBV monoinfection and 22 with HBV–HDV coinfection. Monoinfection was defined as individuals who were HBsAg positive and total anti-HDV negative, while coinfection was defined as individuals who were HBsAg positive and total anti-HDV positive. Indigenous individuals and those coinfected with HIV or HCV were excluded from the study.

### 2.4. Data Collection

All retrospective data collection was conducted through the analysis of electronic medical records. Clinical variables included jaundice, portal hypertension, ascites, cirrhosis, fecal acholia, hepatomegaly, and splenomegaly. Epidemiological data encompassed risk factors for exposure to hepatitis B and hepatitis delta, as well as previous comorbidities and coinfections. Laboratory test results included platelet count, aspartate aminotransferase (AST), alanine aminotransferase (ALT), high-density lipoprotein cholesterol (HDL), alpha-fetoprotein, total bilirubin and its fractions, urea, creatinine, albumin, international normalized ratio (INR), HBsAg, anti-HBs, total anti-HBc, HBeAg, anti-HBe, anti-HDV, and HBV-DNA viral load. At the time of the study, the Brazilian public health system did not provide a test for HDV-RNA viral load.

### 2.5. Indirect Markers of Liver Fibrosis

To estimate the fibrosis stage, the AST to platelet ratio index (APRI) and the fibrosis index for liver fibrosis (FIB-4) were calculated for all patients with available laboratory tests, using the following previously established formulas: APRI = (AST/platelet count) × 100 and FIB‐4 = (age [years] × AST)/(platelet count × 20). The upper limit of normal (ULN) for AST was considered to be 35 IU/L.

### 2.6. Statistical Analysis

Statistical analysis was performed using IBM SPSS Version 25.0, GraphPad PRISM Version 9.0, and Tableau Version 10.5. For categorical variables, chi-squared tests (for large samples) and Fisher's exact test (for small samples) were used to analyze differences. For continuous variables, Student's *t*-test was applied to parametric data, with a *p* value of < 0.05 considered statistically significant.

## 3. Results

**Figure 1:**
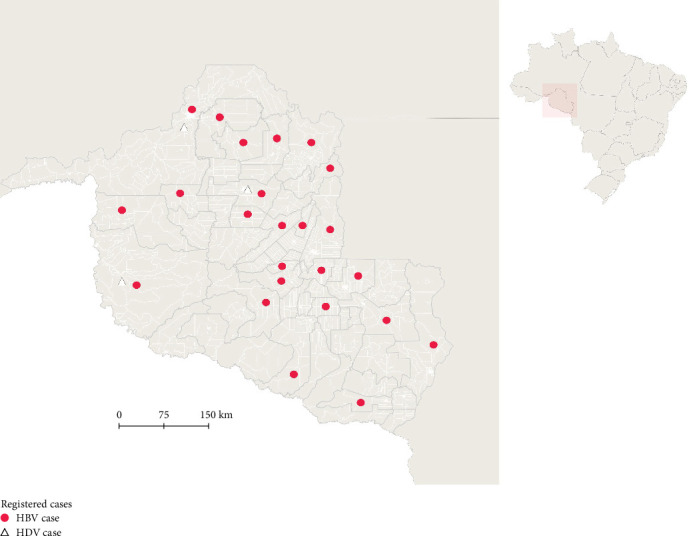
Geographical distribution of HBV and HDV infections in Rondônia monitored by the Viral Hepatitis Outpatient Clinic in Rondônia. The municipalities of Porto Velho, Ariquemes, and Guajará-Mirim had simultaneous records of monoinfected and coinfected patients. The cases were differentiated using symbols.

In this study, 565 individuals were recruited from the AHV/RO outpatient clinic. Of these, 312 were monoinfected with HBV, 22 were coinfected with HDV, and 231 had hepatitis due to other causes. Ten individuals from the HBV monoinfected group were excluded based on the study's inclusion and exclusion criteria. A total of 324 individuals were included in the final analysis, of whom 93.2% (302/324) were infected with HBV and 6.8% (22/324) were coinfected with HDV (Figure 1).

The monoinfected population was widely distributed across the state of Rondônia, covering 46.1% (24/52) of municipalities with registered cases, while only 5.8% (3/52) of municipalities reported cases of coinfection (Figure 1).

Of the 302 monoinfected individuals, the mean age was 46 years (range: 18–77), with a standard deviation of 13.2, and 53.3% (161/302) were male. Among the coinfected individuals, the mean age was 42.8 years (SD = 11.1), and 63.6% (14/22) were male. No statistically significant difference was found between the ages of the two groups (*p* = 0.215). Only 2% (6/324) of participants were 20 years old or younger.

The risk factors for exposure among monoinfected individuals were as follows: 30.5% (92/302) reported having first-degree relatives infected with HBV, 12.9% (39/302) had received a previous blood transfusion, 7.9% (24/302) had tattoos, 3.6% (11/302) were men who reported having sex with other men (MSM), and 3% (9/302) reported the use of intravenous drugs.

In the HDV coinfected population, 40.9% (9/22) of patients reported family contact with HBV carriers, with 9.1% (2/22) also having family contact with HDV carriers. Additionally, 18.2% (4/22) of patients had tattoos, and 9.1% (2/22) were MSM (Table 1).

**Figure 2:**
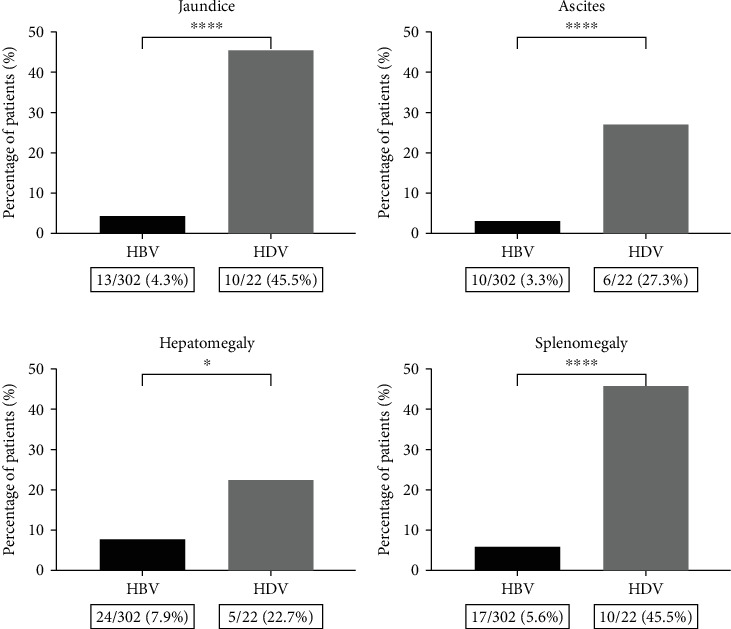
Clinical characteristics of HBV and HBV/HDV patients. (a) Jaundice. (b) Ascites. (c) Hepatomegaly. (d) Splenomegaly. Significant statistical differences are represented in the bars, and the level of significance is expressed as ⁣^∗^*p* < 0.05, ⁣^∗∗^*p* < 0.01, ⁣^∗∗∗^*p* < 0.001, ⁣^∗∗∗∗^*p* < 0.0001, and ns = not significant.

At the first consultation, 16.2% (49/302) of individuals with HBV presented with signs or symptoms of chronic liver disease, compared to 59.1% (13/22) of those with HDV (*p* < 0.0001). Signs of portal hypertension, including ascites, splenomegaly, and visible venous collaterals, were observed in 7.9% (24/302) of HBV patients and 54.5% (12/22) of HDV coinfected patients (Figure 2).

**Figure 3:**
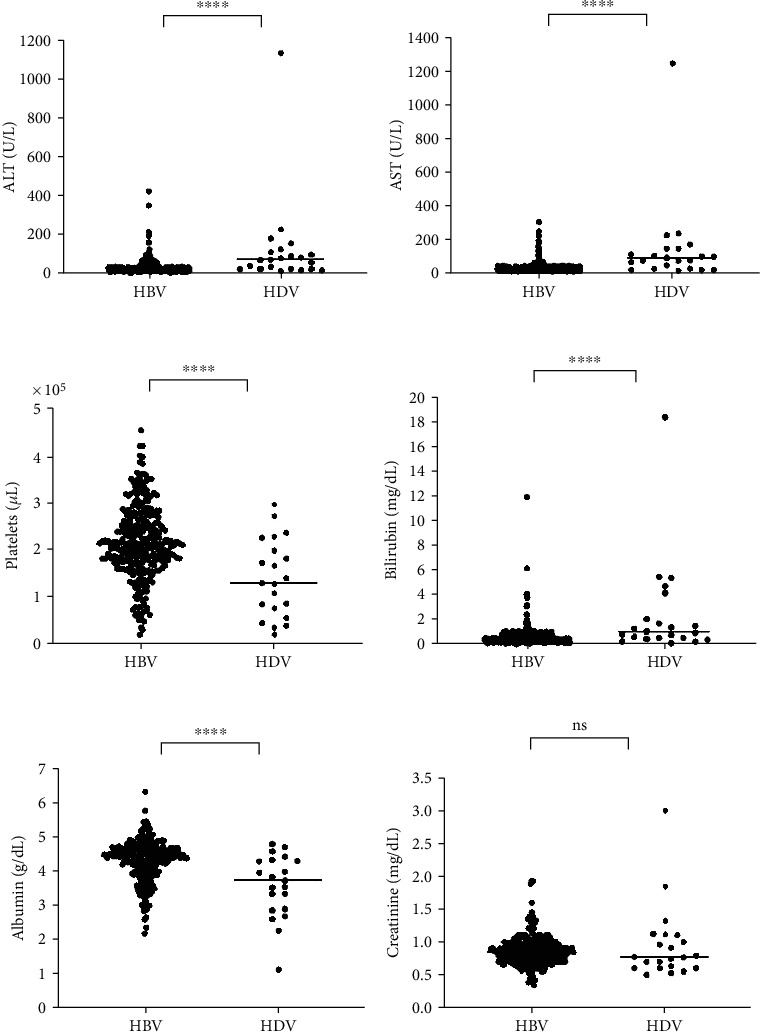
Laboratory tests of HBV and HDV patients. (a) ALT in HBV had a mean of 35.9 (SD = 40.1, range: 5–422); in HDV, the mean was 122.5 (SD = 231.6, range: 15–1128). (b) AST in HBV had a mean of 34.9 (SD = 35.3, range: 10–302); in HDV, the mean was 137.5 (SD = 252.7, range: 17–1,238). (c) Platelets in HBV had a mean of 214.7 (SD = 76.7, range: 21–451); in HDV, the mean was 137.9 (SD = 81.7, range: 21–295). (d) Bilirubin in HBV had a mean of 0.69 (SD = 0.9, range: 0.07–12.0); in HDV, the mean was 2.4 (SD = 4.8, range: 0.01–17.0). (e) Albumin in HBV had a mean of 4.3 (SD = 0.6, range: 2.2–6.3); in HDV, the mean was 3.6 (SD = 0.9, range: 1.2–4.8). (f) Creatinine in HBV had a mean of 0.9 (SD = 0.2, range: 0.4–1.9); in HDV, the mean was 1.0 (SD = 0.5, range: 0.5–3.0). Each dot represents an individual patient, with horizontal bars indicating the mean. Significance is expressed as ⁣^∗∗∗∗^*p* < 0.0001 and ns = not significant.

Significant differences between these groups were observed in markers of liver inflammation (ALT and AST), markers of fibrosis (platelet count), and indicators of decompensated liver function (bilirubin and albumin) (Figure 3).

Viral load assessments were conducted in 293 monoinfected patients, with 3.8% showing undetectable HBV DNA. Among coinfected patients, 95.45% (21/22) had HBV viral load measurements, with 9.5% exhibiting undetectable HBV DNA. However, no significant difference in HBV viral load was observed between the two groups. HBeAg was positive in 6.3% of monoinfected patients and 13.6% of coinfected patients.

**Figure 4:**
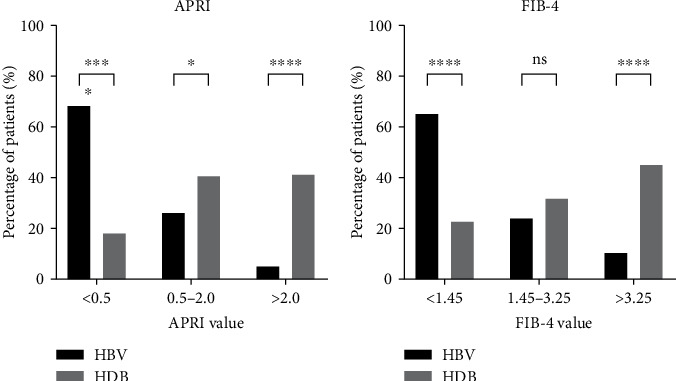
Comparison of hepatic fibrosis in HBV and HDV patients using noninvasive methods with established HBV cut-offs. (a) Aspartate aminotransferase to platelet ratio index. (b) Fibrosis index based on four factors. Significant statistical differences are represented in the bars, and the level of significance is expressed as ⁣^∗^*p* < 0.05, ⁣^∗∗^*p* < 0.01, ⁣^∗∗∗^*p* < 0.001, ⁣^∗∗∗∗^*p* < 0.0001, and ns = not significant.

Biochemical analysis of fibrosis using the APRI and FIB-4 scores revealed statistically significant differences for F0–F1 and F3–F4 (Metavir). Mild fibrosis (F0–F1) was more prevalent in individuals monoinfected with HBV (*p* < 0.0001), while advanced fibrosis (F3–F4) was more common in those coinfected with HBV/HDV (*p* < 0.0001) (Figure 4).

## 4. Discussion

The epidemiology of hepatitis delta is closely linked to the prevalence of HBV in populations, as HDV relies on HBsAg for productive infection in humans [20]. The Brazilian Amazon Basin, comprising the states of Acre, Amazonas, Rondônia, and Roraima, is recognized by multiple studies as an area with high endemicity for HBV and HDV [21, 22]. Therefore, studying the epidemiological and clinical profiles of individuals monoinfected with HBV and coinfected with HDV in these regions is crucial. In Brazil, the epidemiology of HBV and HDV reveals high detection rates in the northern states, particularly in the capitals of Rondônia, Acre, and Roraima [18, 23]. These data support our study, as Rondônia has shown widespread case distribution, including the concurrent circulation of HBV and HDV among infected individuals in the same locations, specifically in the cities of Porto Velho, Guajará-Mirim, and Ariquemes.

The variation in case rates across localities may be associated with the risk of infection through intrahousehold transmission. A study conducted in Amazonas revealed a high proportion of positive serological markers for infection among family members of HBV-infected patients (51.6%) [24]; this is particularly relevant in our study, as approximately 30% of HBV patients reported having a family member also infected with the virus. Among coinfected patients, 45.6% had family contact with HBV, and 9.1% had family contact with HDV. Similar findings were observed in Cameroon, where a ninefold increase in the risk of HDV infection was noted for individuals exposed at home to family members who were carriers [25].

Several studies have reported that hepatitis delta is more aggressive to liver parenchyma than HBV monoinfection, leading to severe clinical manifestations. It is often associated with advanced fibrosis staging and the progression of the pathological condition [26–28]. In our comparative analysis between monoinfected and coinfected individuals, we observed manifestations such as jaundice, ascites, hepatomegaly, and splenomegaly primarily in individuals with hepatitis delta. Laboratory tests revealed altered levels in most coinfected patients, with significant changes observed in liver transaminases (AST and ALT), platelets, bilirubin, and albumin. These findings indicate a greater severity of liver disease caused by HDV.

Among the methods for assessing fibrosis stages, liver biopsy remains the gold standard for this type of investigation [29]. However, due to its invasive nature, liver biopsy is often associated with risks, especially in chronic patients. This is why noninvasive methods for classifying liver disease are crucial [30].

In our study, a comparative analysis between monoinfected and coinfected patients revealed that the APRI and FIB-4 scores indicated values consistent with severe fibrosis, particularly in individuals with hepatitis delta. This confirms that the progression of liver disease caused by HDV is much more severe compared to HBV infection. Scores such as APRI and FIB-4 are essential as noninvasive methods for monitoring liver fibrosis, as they are calculated using commonly available laboratory tests and are widely used in the management of hepatitis B and C [31]. Although hepatic elastography is a highly recommended noninvasive method for assessing liver disease, its high cost poses a significant challenge in regions with limited resources and lower income. It is important to note, however, that there is currently no validated predictive score for assessing fibrosis levels specifically for hepatitis delta.

When evaluating the clinical characteristics of the study population at the first visit, our data indicated that many patients already exhibited signs of advanced liver disease at the time of screening, particularly those with HDV. Among coinfected patients, 45.5% displayed signs of advanced liver disease at the onset of medical follow-up. One study reported that, in HDV coinfection, progression to cirrhosis typically occurs within 5 years, while progression to hepatocellular carcinoma takes an average of 10 years [32]. According to the literature, patients infected with HDV exhibit a more aggressive clinical presentation compared to those monoinfected with HBV alone, with a threefold higher risk of developing cirrhosis [33].

Our study has several important limitations that should be acknowledged. The primary limitation is its retrospective design, which may have introduced registration bias, particularly concerning the risk factors for HBV and HDV infection. Additionally, relevant laboratory tests, such as alpha-fetoprotein and the international normalized ratio of prothrombin time (INR-PT), were not available during the first visit.

## 5. Conclusion

The analysis of epidemiological, virological, and clinical factors is crucial for understanding the pathophysiology and progression of viral hepatitis. In Brazil, hepatitis delta remains a significant public health issue due to its neglected nature and its considerable impact on populations in the Western Amazon region. Our study underscores the clinical importance of investigating hepatitis delta and highlights its more aggressive progression compared to hepatitis B. Additionally, our research emphasizes the need for testing individuals who are HBV positive, as our findings show the simultaneous presence of both HBV and HDV in neighboring regions of Rondônia, as well as in Amazonas and Acre states. We also stress the importance of further studies exploring the molecular aspects of hepatitis delta and the factors influencing the disease's pathophysiology.

## Figures and Tables

**Table 1:** Clinical–epidemiological characteristics of HBV and HDV patients in 2017 and 2018.

	**HBV** **n** = 302	**HDV** **n** = 22	**p**
*Epidemiological data*			
Age (years), average (min–max)	46 (18–77)	42.8 (28–77)	0.292
Male, *n* (%)	161 (53.3)	14 (63.6)	0.348
*Ethnicity, n (%)*			
Mixed race	257 (85.1)	21 (95.5)	0.179
White	24 (7.9)	0 (0)	0.169
Black	13 (4.3)	1 (4.5)	0.957
Not reported	8 (2.7)	0 (0)	0.439
*Comorbidities, n (%)*			
Previous case of malaria	183 (60.6)	14 (63.6)	0.778
SAH	49 (16.2)	1 (4.5)	0.143
Diabetes mellitus	24 (7.9)	0 (0)	0.169
*Risk factors, n (%)*			
Family history of HBV	93 (30.8)	10 (45.6)	0.154
Transfusion	39 (12.9)	1 (4.5)	0.249
Tattoo	24 (7.9)	4 (18.2)	0.099
MSM	11 (3.6)	2 (9.1)	0.209
IVDU	9 (3)	1 (4.5)	0.682
Hemodialysis	4 (1.3)	0 (0)	0.587
Family history of HDV	0 (0)	2 (9.1)	< 0.0001

*Note: n*, number of patients evaluated; max, maximum; min, minimum.

Abbreviations: HBV, hepatitis B virus; HDV, hepatitis delta virus; IVDU, intravenous drug user; MSM, men who have sex with men; SAH, systemic arterial hypertension.

## Data Availability

The data used and analyzed in this study are available upon request from the corresponding author. If necessary, metadata and supporting information can be provided for the purpose of reproducing and validating the reported results.
